# Spectrum Sensing in Very Low SNR Environment Using Multi-Scale Temporal Correlation Perception with Residual Attention

**DOI:** 10.3390/s25020528

**Published:** 2025-01-17

**Authors:** Song Hong, Weiqiang Xu

**Affiliations:** 1 School of Computer Science and Technology, Zhejiang Sci-Tech University, Hangzhou 310018, China; 202220601001@mails.zstu.edu.cn; 2 School of Information Science and Engineering, Zhejiang Sci-Tech University, Hangzhou 310018, China

**Keywords:** spectrum sensing, multi-scale, correlation, attention, deep learning

## Abstract

Spectrum sensing is recognized as a viable strategy to alleviate the scarcity of spectrum resources and to optimize their usage. In this paper, considering the time-varying characteristics and the dependence on various timescales within a time series of samples composed of in-phase (I) and quadrature (Q) component signals, we propose a multi-scale time-correlated perceptual attention model named MSTC-PANet. The model consists of multiple parallel temporal correlation perceptual attention (TCPA) modules, enabling us to extract features at different timescales and identify dependencies among features across various timescales. Our simulations show that MSTC-PANet significantly improves the detection of channel occupancy at low signal-to-noise ratios (SNR), particularly in untrained scenarios with lower SNR conditions and modulation uncertainties. The analysis of the ROC curve indicates that at an SNR of -20 dB, the proposed MSTC-PANet achieves a detection rate of 98% with a false alarm rate of 10%. Furthermore, MSTC-PANet, which has been trained using digital modulation techniques, also demonstrates applicability to analog modulation.

## 1. Introduction

The rapid advancement of radio technology has led to the significant utilization of wireless devices in emerging application domains, making spectrum resources increasingly scarce. The increasing demand for more effective and complex communications underscores the critical importance of spectrum resources as the foundation for wireless communication systems [1]. Nevertheless, the conventional fixed resource allocation approach predominantly reserves spectrum resources for licensed primary users, leaving only a fraction available for unlicensed secondary users [2,3]. Many existing works have proposed various spectrum sensing methods to address the issue of low spectrum resource utilization caused by traditional fixed resource allocation methods.

Various traditional spectrum sensing methods have been developed to address the challenges of detecting primary users (PUs) in cognitive radio networks. Matched filtering [4] is effective in scenarios with high SNR but requires prior knowledge of the PU signal and involves complex computations. Energy detection (ED) [5] does not require prior information about the PU signal but is prone to high false alarm rates and low detection probability at low SNR levels. Covariance absolute value detection (CAV) [6] leverages statistical covariance disparities between the signal and noise to determine channel occupancy. Cross-Correlation Absolute Value Detection (SCCAV) [7] utilizes the absolute value of the statistical cross-correlation of data received from multiple antennas to construct a test statistic. This method significantly enhances the detection performance by aggregating the correlation between signals. Maximum eigenvalue detection (MED) [8] and cyclostationary feature detection (CFD) [9] involves analyzing the maximum eigenvalue of the signal sample covariance matrix and the general correlation function of the signal, respectively. In addition, the 3EDD algorithm [10], which is based on energy detection, employs Newton’s method to expedite the search for an accurate approximation of the optimal decision threshold that minimizes the decision error probability. The enhanced collaborative energy detection method builds upon traditional energy detection techniques, significantly improving detection accuracy by integrating information such as frequency, phase, and amplitude. The techniques have undergone enhancements in recent decades, partially meeting the pressing demand for spectrum resources. Nevertheless, conventional methods depend on particular assumptions for achieving optimal performance, potentially making these models unsuitable and resulting in performance deterioration in the event of environmental changes.

In recent years, the advanced feature extraction capabilities of deep learning have led to significant advancements in spectrum sensing. In [11], Kai et al. proposed a deep neural network integrated with LSTM and analyzed the impact of various LSTM layers on detection performance. In [12], a blind feature extraction method utilizes capsule networks (CAP) and cyclic cumulant (CC) features of the signal to classify digitally modulated signals. In [13], Chen et al. present a short-time Fourier transform-based convolutional neural network (STFT-CNN) to utilize time-varying information in the frequency domain. In [14], Lees et al. proposed a Short-Time Fourier Transform (STFT)-based perception method for broadband spectrum sensing. This method utilizes each time slice of the broadband frequency band to compute the STFT and generate matrix data for classification. Furthermore, several studies focus on utilizing deep learning techniques for spectrum sensing by leveraging spectral correlation functions. These models use spectrum correlation functions as inputs, demonstrating superior performance. For instance, in [15], Tekbıyık et al. utilize spectral correlation functions for spectrum sensing and signal identification. Additionally, in [16], Chae et al. propose a DS2MA model that conducted experiments using cross-correlation between multiple antennas and the internal correlation of a single antenna. Moreover, in [17], Liao et al. proposed an improved Gaussian mixture model that utilizes a three-dimensional feature extraction method. This method leverages the complementarity of signals in both the time and frequency domains to construct fusion features, which are then used to train the model.

Existing spectrum sensing methods mainly focus on studying the single time scale relationship within the time series, or combining the correlation between multiple different dimensions [17], such as the frequency domain and the time domain. Among the above methods, the cross-correlation function, LSTM network, and STFT network have shown extraordinary potential in capturing the temporal dynamics of a single scale within the sequence [13,18]. At the same time, converting multivariate time series into two-dimensional matrix data also provides a new perspective for studying multivariate time series [16]. However, for existing spectrum sensing methods, there is a major oversight of the changes in the correlation between sequences at multiple different time scales in the time series, and the correlation between different time scales within the time series has not been well studied. In addition, more and more studies have shown that there are inseparable correlations between multiple time scales. These correlations have been applied in various fields and have achieved remarkable results in traffic forecasting in [19], temperature forecasting in [20], and biological population change forecasting in [21].

In the above examples, the limitations of traditional deep learning are obvious, because they usually cannot capture the various dependencies between the variables under consideration and the correlations that change over time [22]. For example, the DSS model [23] calculated using the autocorrelation function cannot perform reasonable calculations on different time scales and cannot make timely adjustments to the state of environmental changes. For the above reasons, we studied the correlation between different time scales of time series and the connection between time changes, and applied it to the field of spectrum sensing, achieving remarkable results.

In order to overcome the limitations of existing deep learning models and further utilize the correlation between different scales of time series, we propose a multi-scale time-correlated perceptual attention model named MSTC-PANet. This model employs a parallel structural design to leverage the correlations both within and between different scales of time series data. This study explores the correlation between IQ sequences of spectral sequences at multiple different time scales and considers the effectiveness of correlation functions in extracting time delay features in dynamic environments. In addition, we design a convolutional layer dedicated to learning time scale correlation and use a multi-head attention mechanism to capture the correlation of the current time scale. The main contributions of this work are as follows:

1. To enhance the precision of channel perception under conditions of low SNR, we proposed the MSTC-PANet. Furthermore, we designed the TCPA module with a unique architecture to facilitate efficient feature extraction at a specific time scale.
2. To achieve multi-timescale perception and identify the dependencies at different timescales, we designed a parallel input structure consisting of multiple TCPA modules. This parallel structure can effectively integrate the correlations at different timescales and significantly improve the classification accuracy.
3. The model can autonomously learn correlations from IQ sequences across various timescales, enabling the efficient extraction of feature information from untrained modulation patterns. Simulations demonstrated that our proposed MSTC-PANet exhibits excellent detection performance and strong resilience to noise at low SNR.

## 2. Problem Formulation

### 2.1. Signal Model

In cognitive radio (CR), accurately detecting spectrum occupancy is essential. To improve the precision of channel occupancy detection, we investigated non-cooperative spectrum sensing within a single frequency band. While the detection method utilized by a single sensing node in a single frequency band can be adapted for multi-node sensing scenarios, our primary focus remains on the single sensing node. The process of detecting the presence of a signal in a radio spectrum is treated as a problem of binary hypothesis testing [24], where $H_{1}$ represents the channel currently being occupied by PU, and $H_{0}$ denotes that the channel is not currently occupied by PU. As a result, in any detection cycle, the signal sampling of SU can be expressed as follows [25]:

(1) $$r\left(n\right)=\left\{\begin{array}{cc} h(n)s(n)+w(n) & H_{1}\\ w(n) & H_{0} \end{array}\right.$$

where r(n) represents the *n*th signal sampling of SU, with n=1,2,3…,N. *N* is the length of the sample. s(n) is the modulation signal transmitted on the current frequency band if it is occupied by a certain PU. h(n) represents the channel gain. w(n) represents additive Gaussian noise with a mean of zero and variance $\sigma_{w}^{2}$.

After obtaining the sampled complex data, which consist of in-phase (I) and quadrature (Q) components. The I component and Q component are extracted into vectors of dimension 1xn, respectively, and then combined into a matrix. Consequently, the processed data of received signal samples can be expressed as follows:

(2) $$R={\left[\begin{array}{cc} r_{I} & r_{Q} \end{array}\right]}^{T}$$

where R represents a matrix composed of the I and Q components, with dimensions 2×N. The vectors $r_{I}$ and $r_{Q}$ correspond to the real and imaginary components, respectively. T represents the transpose of a matrix.

To remove the dependence on the power information of the neural network, the average power of R is normalized to 1. The specific normalization implementation is as follows:

(3) $$X=\frac{R}{\sqrt{\frac{1}{N}\left(∥r_{I}{∥}^{2}+{∥r_{Q}∥}^{2}\right)}}$$

where X represents the average power normalized data for the sampled data, and ${∥\cdot ∥}^{2}$ represents the square of the vector modulus length.

### 2.2. Multi-Time Scale Correlation

Despite the notable interconnections among various temporal scales within a time series, conventional deep neural networks often struggle to effectively capture these relationships. The existing literature [26,27,28] consistently demonstrates the correlations present across different temporal scales in time series data. Consequently, in contexts characterized by uncertain temporal relationships, aggregating multiple time scales can enhance the ability of a model to learn more complex features. In the field of spectrum sensing, spectrum sequences exhibit pronounced periodicity and correlation within the temporal dimension. Given this context, an investigation into the internal correlations of spectrum sequences is conducted to foster a deeper understanding of the inherent interrelationships within these sequences. Furthermore, the correlations and collaborations present within the sequence contribute to the model’s robustness in challenging environments characterized by high noise levels, ultimately facilitating faster model convergence.

**Figure 1:**
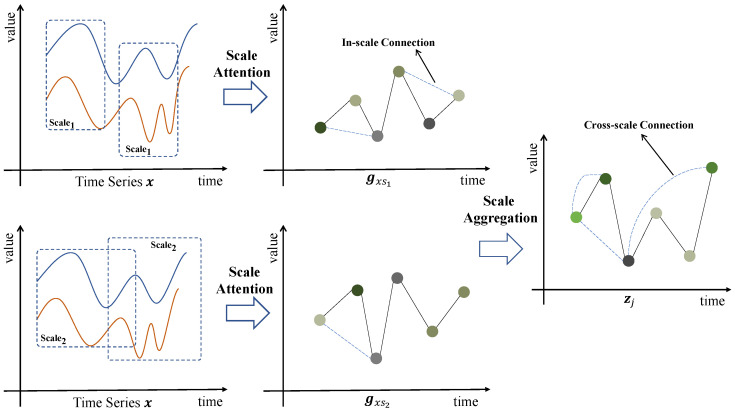
Scale Attention and Relation.

Figure 1 depicts a scenario characterized by two distinct temporal scales. The left section of the figure presents a sampled time series, denoted as x, which is analyzed across these two different time scales. The upper portion illustrates observations corresponding to the shorter time scale, referred to as $Scale_{1}$, while the lower portion details observations associated with the longer time scale, designated as $Scale_{2}$. The cross-correlation results of this time series with another sequence, y, are computed at both time scales. In the figure, the term “Scale Attention” denotes the computation of the aforementioned cross-correlation, alongside the application of Attention mechanisms to ascertain correlations within the sequence. This component is analogous to the TCPA module that we have developed. It is crucial to emphasize that a singular observation at a specific scale can only yield correlations within that scale and is incapable of capturing correlations across different scales. To leverage the inter-scale correlations, we implement a feature fusion process via Scale Aggregation, which involves the direct summation of values across corresponding dimensions. In the figure, positive features are indicated in green, whereas negative features are depicted in gray. Notably, positive features identified at a shorter time scale may either diminish or intensify when assessed at a longer time scale. Conversely, negative features observed at a shorter scale may manifest as positive features in a longer scale context [21]. The dotted lines in the figure signify correlations that exist within individual scales. Following feature aggregation, connections can be established between disparate time scales. Ultimately, the aggregation of features across these various temporal scales can substantially mitigate the limitations inherent in neural networks, thereby enhancing their capacity to recognize more distant relationships and establish more intricate connections.

Signal correlation analysis is a technique employed to assess the degree of similarity between a signal and itself or with other signals at a specific time lag. In the case of discrete signals denoted as x[n] and y[n], the cross-correlation function is formally defined as follows:

(4) $$R_{xy}\left[\tau \right]=\sum_{n=0}^{N-1}x\left[n\right]y^{*}\left[n+\tau \right]$$

where $R_{xy}\left[\tau \right]$ represents the similarity of signals x[n] and y[n] at delay τ, τ=0,1,2,…,N−1. $y^{*}\left[n\right]$ is the conjugate of y[n].

Before Scale Aggregation, we perform cross-correlation calculations on the time series x and the sequences $S=\{s_{1},s_{2},s_{3},\ldots ,s_{n}\}$ of varying lengths. Consequently, $G_{xs}$ operates as a single scale within a sequence while demonstrating multiple scales across different sequences.

(5) $$g_{xs_{i}}=\left[\begin{array}{ccccc} R_{xs_{i}}\left[1\right] & R_{xs_{i}}\left[2\right] & R_{xs_{i}}\left[3\right] & \cdots & R_{xs_{i}}\left[n\right] \end{array}\right]$$

(6) $$G_{xs}=\left\{g_{xs_{1}},\;g_{xs_{2}},\;g_{xs_{3}},\;\cdots ,\;g_{xs_{n}}\right\}$$

where $s_{i}$ represents the *i*-th learnable sequence, with a length of $Scale_{i}$, and $g_{xs_{i}}$ denotes the value of the cross-correlation function calculated between x and $s_{i}$.

We sum each item in the result set $G_{xs}$ to capture the correlation across different time scales. This step can be expressed as follows:

(7) $$z_{j}\;=\;\sum_{i=1}^{n}g_{xs_{i}}\left[j\right]$$

(8) $$Z=\left[\begin{array}{ccccc} z_{1} & z_{2} & z_{3} & \cdots & z_{n} \end{array}\right]$$

where $z_{j}$ is the *j*-th dimension of the scale aggregation result Z.

Since the calculation of the cross-correlation function can be replaced by convolution, we integrated a one-dimensional convolution layer into our network as a functional convolution layer. Furthermore, to adapt to the correlation changes caused by variations in the IQ sequence over time, we adjusted the convolution kernel size of the functional convolution layer to compute the correlation of IQ sequences at different timescales. The objective of this approach is to enable the neural network to effectively learn each sequence within the designated set S, which represents the one-dimensional parameter sequence of each functional convolutional layer participating in the parallel network.

## 3. MSTC-PANet Design

### 3.1. Network Architecture Design

**Figure 2:**
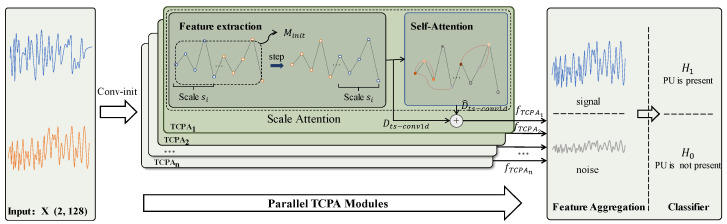
Structure of MSTC-PANet.

Since signals are susceptible to changes in the environment and exhibit time-varying characteristics, IQ sequences will exhibit changes in amplitude or phase. To capture the time-varying nature and take advantage of correlations at different timescales, we propose MSTC-PANet to make up for the shortcomings of the existing spectrum sensing framework. The structure of our proposed MSTC-PANet is depicted in Figure 2.

This model consists of multiple parallel TCPA modules, with each TCPA module responsible for extracting the correlation of IQ sequences at different timescales. The parallel TCPA module contains four processing steps in total: (1) identifying the time scale of the input sequence, (2) capturing the intra-sequence correlation through the attention mechanism, (3) capturing the correlation between the captured sequence and the original sequence through residual connection, and (4) capturing the correlation between different time scales through feature aggregation. This innovative approach helps extract correlations at different time scales, which helps to attenuate the noise portion of the signal and identify hidden features in time-varying signals. Table 1 provides the parameters of MSTC-PANet.

First, to enhance the dimensional information of the original data, the energy-normalized input data X are passed through the initialization convolution layer, corresponding to the Conv-init operation depicted in Figure 2. This layer comprises a Conv1d layer and a BatchNorm1d layer. This process can be expressed as follows:

(9) $$M_{init}=f_{init}\left(X\right)$$

Following the initialization of the feature data $M_{init}$, they are concurrently input into multiple parallel TCPA modules. The next section provides a detailed explanation of the architecture of the TCPA module. Subsequently, the results from these parallel TCPA modules are consolidated. These procedures help the model acquire feature information across different timescales and comprehend the interdependencies of features at various timescales to enhance locally effective features and reduce the impact of noise. This process can be described as follows:

(10) $$Z\;=\;\sum_{k=1}^{K}f_{TCPA_{k}}\left(M_{init}\right)$$

where *K* represents the number of defined TCPA modules, and $f_{TCPA_{k}}\left(M_{init}\right)$ represents the operation of the *k*th TCPA module.

Finally, we construct an output layer comprising one Dense layer to accurately classify features. We flatten the last two dimensions of the embedded data Z into $\tilde{z}$, which is then passed through a linear transformation and a Softmax function to derive the probability y for each feature. This process can be expressed as follows:

(11) $$y\;=\;Softmax(W_{1}\tilde{z}+b)$$

The final output y is the respective value of $P_{H_{1}}\left(X|\theta \right)$ and $P_{H_{0}}\left(X|\theta \right)$ when the input data are X where $P_{H_{1}}\left(X|\theta \right)+P_{H_{0}}\left(X|\theta \right)=1$.

### 3.2. Temporal Correlation Perceptual Attention (TCPA) Module

**Figure 3:**
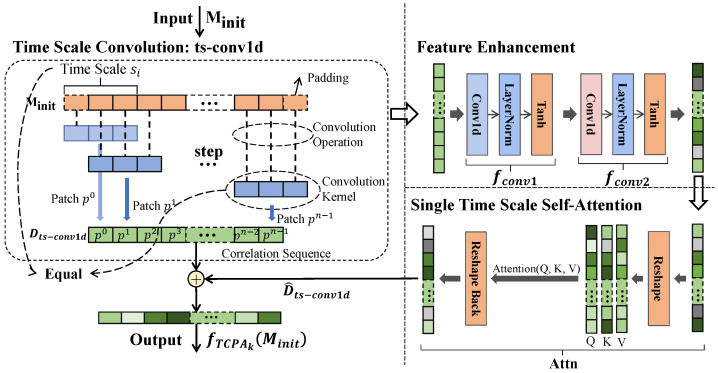
Structure of Temporal Correlation Perceptual Attention (TCPA) Module.

Figure 3 shows the structure of the TCPA module. The parameters of the TCPA module are outlined in Table 2. To effectively capture features across various time scales, the TCPA module employs a convolutional layer specifically designed for time-scale convolution, referred to as ts-conv1d.

As illustrated in Figure 3, during the processing of the *i*-th TCPA module, the scale $s_{i}$ is utilized to traverse the time series. The blue square section in the figure represents the convolution kernel of ts-conv1d, while “step” denotes the traversal operation. The length of the convolution kernel in ts-conv1d is equal to the length of the time scale $s_{i}$ that is to be extracted. After the sequence $M_{init}$ is convolved with ts-conv1d, the outcome of the convolution operation for each time series of length $s_{i}$ using the convolution kernel is defined as a “Patch” [22]. Then, the patch $p^{t}$ at time *t* can be expressed as follows:

(12) $$p^{t}=\sum_{c=1}^{C}\sum_{n=1}^{s_{i}}M_{init}^{c}\left(t+n\right)\cdot w_{c}\left(n\right)$$

where *C* is the number of channels, and $M_{init}^{c}\left(t\right)$ represents the value of the time series at channel *c* and time *t*. $w_{c}$ represents the convolution kernel corresponding to channel *c* with the same size as the time scale.

The process of Time Scale Convolution effectively integrates the $p^{t}$ values obtained at each moment *t* into a new sequence, resulting in a correlation sequence that corresponds to the current time scale. The outcome of the Time Scale Convolution can be expressed as follows:

(13) $$D_{ts-conv1d}=\left[\begin{array}{ccccc} p^{0} & p^{1} & p^{2} & \ldots & p^{n-1} \end{array}\right]$$

This layer allows for real-time adjustments to the size of the convolution kernel, enabling the calculation of different time scales. Consequently, the module can perform correlation calculations across various time scales by modifying the size of the convolution kernel designated for time-scale convolution. Compared to traditional methods, this approach facilitates the convenient selection of the desired time scale, the combination of different time scales, and integration into the backpropagation process of MSTC-PANet, thereby enhancing the identification of correlations both within and between time scales.

The initial time-related features, denoted as $D_{ts-conv1d}$, are susceptible to noise, which can obscure a significant number of genuine features. To address this issue, $D_{ts-conv1d}$ is processed through a deep feature extraction module consisting of two convolutional layers with identical structures. Subsequently, the output is fed into a multi-head attention module to capture the correlations among features across the time scale. As illustrated in the Figure 3, the data are transformed into key vectors (K), query vectors (Q), and value vectors (V) within the attention mechanism via a reshape layer, followed by the application of self-attention. This process is defined as follows:

(14) $${\hat{D}}_{ts-conv1d}=Attn\left(f_{conv2}\left(f_{conv1}\left(D_{ts-conv1d}\right)\right)\right)$$

Finally, we have adopted residual connections to apply the features extracted by the deep feature extraction module to the original features to achieve effective feature enhancement and weakening of noise features. In Figure 3, the internal operation of the shortcut is to maintain the dimensional consistency of the backbone data. This method enhances the fitting ability and noise resistance of the neural network, amplifies the meaningful features and reduces the impact of irrelevant features. The processing can be represented as follows:

(15) $$f_{TCPA_{k}}\left(M_{init}\right)\;=\;D_{ts-conv1d}+{\hat{D}}_{ts-conv1d}$$

where $f_{TCPA_{k}}\left(M_{init}\right)$ represents the output of the *k*th TCPA module, which is the valid feature extracted at the current timescale.

Our model can improve our perception accuracy and reduce the rates of false detection and false alarms by constructing multiple parallel TCPA modules. Section 4.4 discusses the sensing error and average classification time on the validation set using different numbers and sizes of variable convolution kernels. It is important to emphasize that the sensing error of the validation dataset decreases as the number of the TCPA increases. However, considering the computational complexity, we use three TCPA modules, with variable convolution kernel sizes of 3, 5, and 7 for each module.

## 4. Numerical Simulations and Analysis

### 4.1. Data Generation

The datasets in this paper were generated using the methods described in [29]. We generate simulation datasets for various digital and analog modulation schemes, including BPSK, 8PSK, QPSK, 16QAM, 64QAM, WBFM, and AM-DSB, using the code provided in the RML2016.10a public dataset. In the specified SNR interval of [−25, 5] dB, a total of 10,000 signal samples were gathered for every modulation scheme under each SNR circumstance, with a 1-dB interval. Each signal sample consisted of 128 IQ samples. In order to adapt to dynamic changes in noise intensity, the training dataset was constructed by blending solely BPSK modulation data falling within the range of [−15, 5] dB, resulting in 4000 training instances per SNR level. The parameters of the dataset generation are shown in Table 3.

### 4.2. Experiment Results

In this section, we have presented a significant number of simulations to compare our proposed MSTC-PANet with CLDNN [30], STFT-CNN [13], and DSS [23]. To evaluate the efficacy and robustness of MSTC-PANet, we utilized the metrics of sensing error [18] and Receiver Operating Characteristic (ROC) as evaluation criteria. Furthermore, by reducing the length of the sampled data to 64, we retrain our model to assess the extent of performance decline resulting from this reduction in sampling length. This process demonstrates the effective feature extraction capabilities and robust noise resistance of the MSTC-PANet, particularly in the context of multi-scale features.

The sensing error is calculated as the average of the false alarm probability and the missed detection probability. The definition of sensing error is as follows:

(16) $$Sensing\;Error\;=\;\frac{P_{fa}+P_{m}}{2}$$

where $P_{m}$ represents the probability that the channel is detected as empty when the PU exists, and $P_{fa}$ represents the probability that the channel is detected as occupied when the PU does not exist. A well-performing model should have low $P_{fa}$ and $P_{m}$.

**Figure 4:**
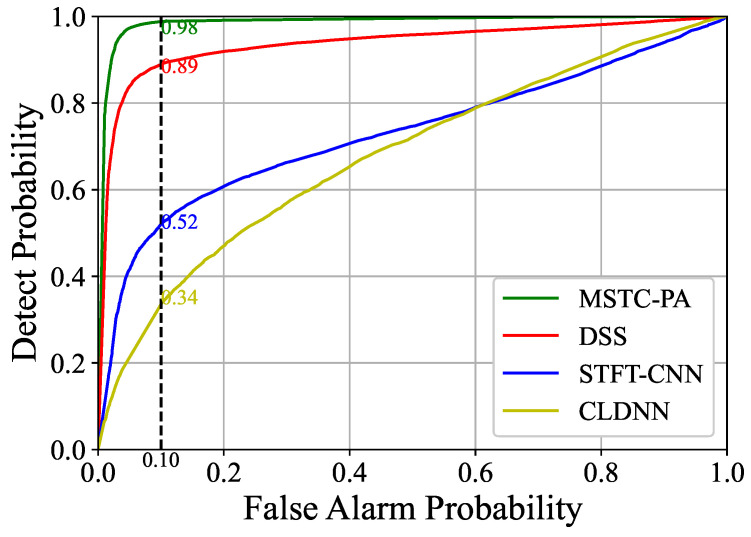
Comparisons of ROC curves for BPSK at −20 dB.

The ROC curves of different network models were analyzed at –20 dB using the Monte Carlo method, as shown in Figure 4. The relationship between $P_{d}$ and $P_{fa}$ varied from 0 to 1. Compared with other models, our MSTC-PANet can maintain a higher Pd under the condition of lower Pfa, which means that our model can clearly distinguish between the cases where PU exists and the cases where PU does not exist, and make more reliable judgments. This also demonstrates that the multi-scale spectrum sensing model with a parallel structure we proposed can extract correlations both within and between different time scales. This enhancement increases the model’s resistance to noise and enables a clearer distinction between the characteristics of signals and noise.

**Figure 5:**
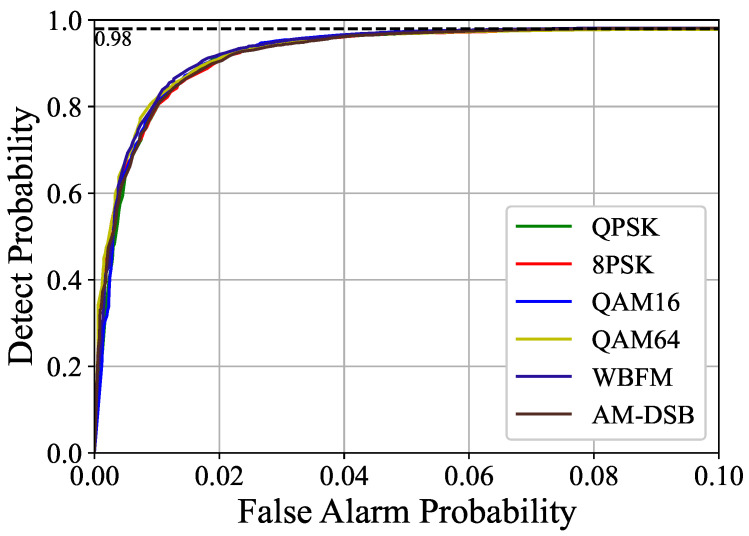
Comparison of ROC curves for QPSK, 8PSK, QAM16, QAM64, WBFM, AM-DSB and QAM16 of MSTC-PANet at −20dB.

Additionally, we evaluated the ROC curve of the MATC-PANet when applied to various modulation modes at −20 dB. Since the $P_{d}$ of the model approaches 1 when the $P_{fa}$ is 0.1, we present the relationship between $P_{d}$ and $P_{fa}$ only within the range of 0 to 0.1. As illustrated in Figure 5, our model consistently achieves a high detection probability for both digital and analog modulation at very low Pfa levels, effectively meeting practical requirements.

**Figure 6:**
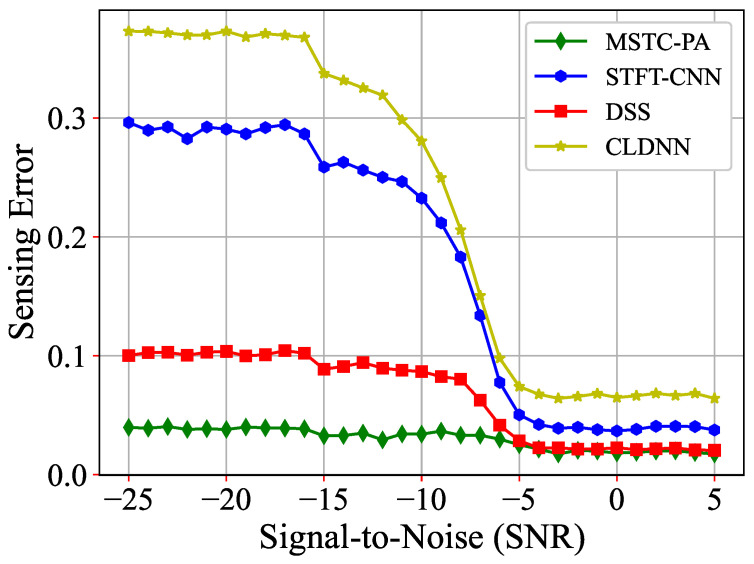
Comparisons of sensing errors for BPSK.

Figure 6 illustrates the comparison of sensing errors between different models, all trained under BPSK modulation. The results indicate a significant increase in sensing errors for all models at −6 dB SNR. Particularly, the sensing error of our proposed MSTC-PANet shows a relatively modest increase from 0.017 at 5 dB to 0.04 at −25 dB. At the SNR of −25 dB, the stable sensing error of MSTC-PANet is 0.04, approximately 40% of DSS, 13% of STFT-CNN and 11% of CLDNN. Compared to other models, MSTC-PANet exhibits a significant advantage in sensing error, demonstrating superior adaptability to high noise levels and robust performance on untrained datasets within the SNR range of −25 dB to −16 dB.

**Figure 7:**
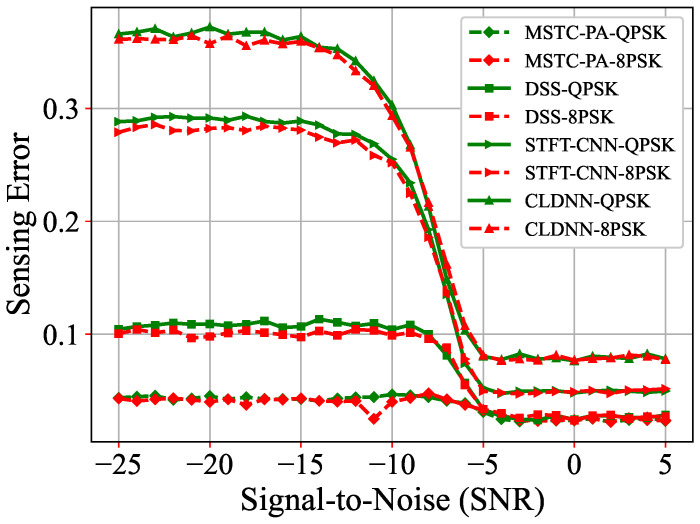
Comparisons of sensing errors for QPSK and 8PSK.

**Figure 8:**
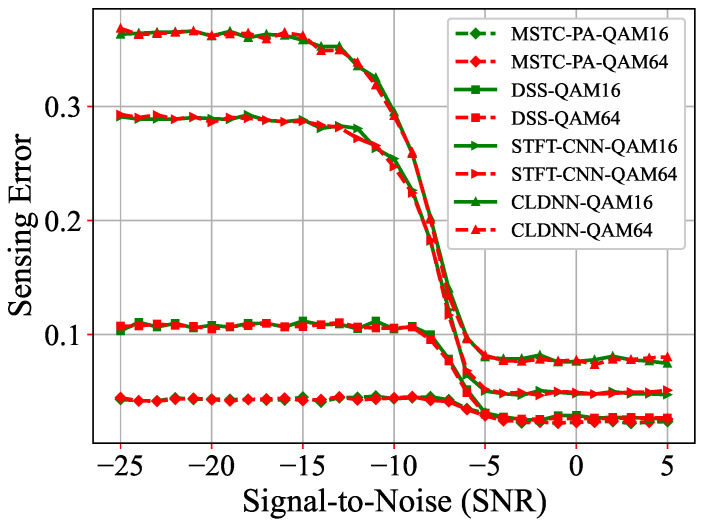
Comparisons of sensing errors for QAM16 and QAM64.

In addition, the performance of the model trained using BPSK is evaluated on other modulation schemes, including 8PSK, QPSK, QAM16, and QAM64, to assess the model’s generalization ability. As shown in Figure 7 and Figure 8, the test results for QPSK, 8PSK, QAM16, and QAM64 are still very satisfactory. Specifically, under conditions of −25 dB, the sensing error of our proposed MSTC-PANet is approximately 0.045, which does not fluctuate significantly when compared to the test results obtained under BPSK modulation. The experimental findings indicate that our MSTC-PANet demonstrates robust adaptability across various modulation types. Notably, the model trained using BPSK can be directly applied to other modulation schemes, thereby reducing the need for retraining the model.

**Figure 9:**
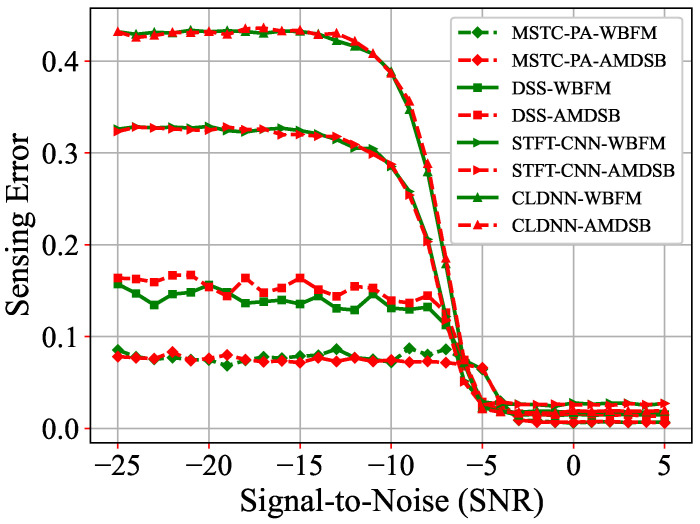
Comparisons of sensing errors for WBFM and AM-DSB.

Furthermore, we applied the model trained on digitally modulated signals to analog modulated signals and compared the sensing errors within the range of –25 dB to 5 dB. Figure 9 illustrates the sensing error associated with a model trained using BPSK digital modulation when applied to two analog modulation techniques: WBFM and AM-DSB. The results indicate that the model trained on digitally modulated signals demonstrates strong adaptability to analog modulation methods. While all models experience varying degrees of performance decline, the proposed MATC-PANet consistently demonstrates the lowest perceptual error while maintaining a relatively stable performance level. The significant fluctuations observed in the DSS and MSTC-PANet can be attributed to the superior noise immunity of digital modulation, whereas analog modulation is more susceptible to noise. Since the primary focus of the research on MSTC-PANet and DSS is the scale characteristics of signal manifestation, the time scale characteristics are more easily obscured by noise, complicating their extraction.

**Figure 10:**
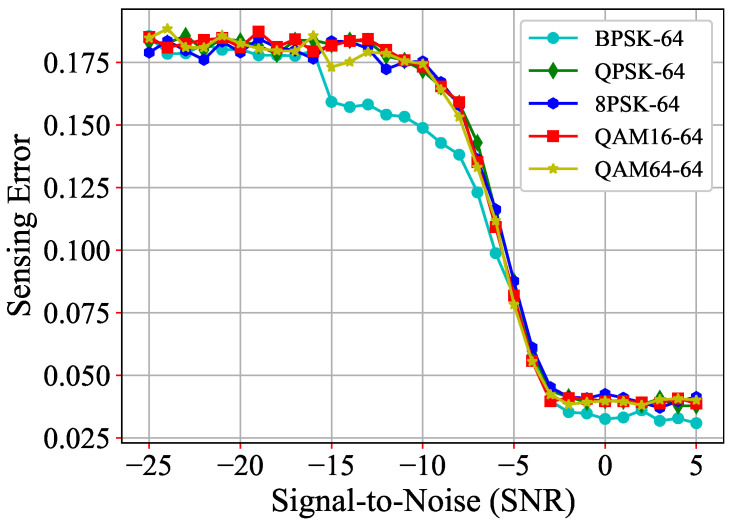
Comparisons of sensing errors for various modulations of MSTC-PANet.

Finally, we reduced the sampling length to 64 to retrain MSTC-PANet while keeping all other conditions unchanged. As illustrated in Figure 10, we evaluated the sensing error of MSTC-PANet using various modulation schemes, including BPSK, QPSK, 8PSK, QAM16, and QAM64. The results indicate that the sensing error of MSTC-PANet fluctuates around 0.185, which is comparable to the performance of DSS and STFT-CNN under a sampling length of 128. These findings demonstrate that even with a reduced sampling length, the proposed MSTC-PANet can still achieve an acceptable level of sensing error, showcasing its strong adaptability.

### 4.3. t-SNE

t-Distributed Stochastic Neighbor Embedding (t-SNE) is a nonlinear technique used for dimensionality reduction and visualization of high-dimensional data. This method effectively preserves the local structure of high-dimensional data when represented in a lower-dimensional space. In the context of neural networks, high-dimensional data are often condensed into two-dimensional or three-dimensional representations to improve the visualization of the internal structures and patterns inherent in the data.

**Figure 11:**
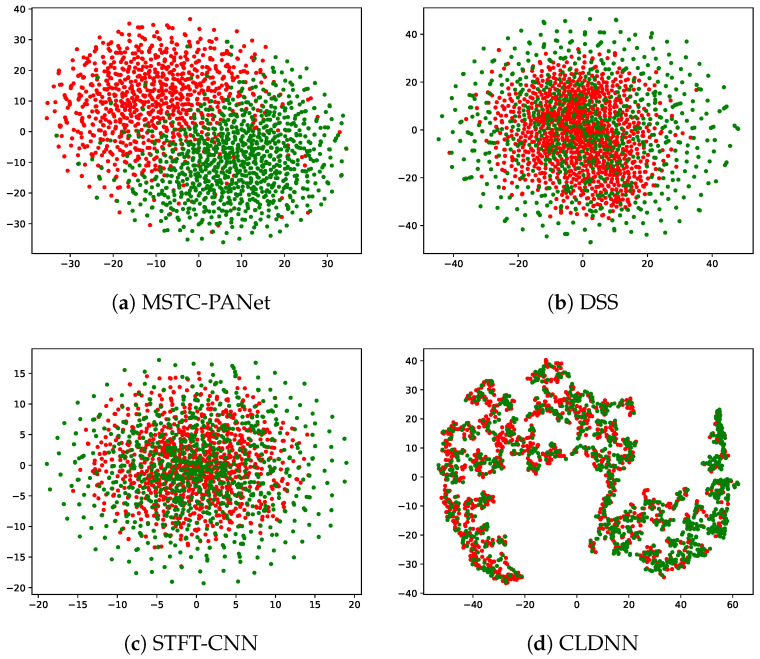
t-SNE results at −25 dB. Green dots are feature visualizations when PU signals are present, and red dots are feature visualizations when PU signals are absent.

Figure 11 illustrates the t-SNE visualizations of both the proposed model and an existing model. The t-SNE method is employed to visualize the features that traverse the feature extraction layer, which includes all layers preceding the Dense layer. This visualization demonstrates the clustering of samples associated with different labels. Green dots represent the presence of PU signals, and the red dots signify the absence of PU signals. The results indicate that the t-SNE representation of the proposed MSTC-PANet effectively differentiates between cases in which the PU is present and those in which the PU is absent, demonstrating greater effectiveness than existing methods. Notably, prior to the feature mapping conducted by the Dense layer, the MSTC-PANet exhibits a clear delineation of the clustering boundaries for various labels. This observation suggests that the MSTC-PANet is proficient in extracting common features from the received signals, facilitating a significant distinction between signals and noise, and identifying critical features that enable the separation of these two categories.

Furthermore, the figure illustrates that the distribution of green points, which represent the DSS model in the presence of PU signals, is more uniform compared to the distribution of red points that signify noise. This observation indicates that the signals exhibit pronounced correlation characteristics, while the noise is characterized by an irregular concentration. In contrast, prior to the feature mapping performed by the Dense layer, the DSS and STFT-CNN models did not achieve a clear distinction of the boundary between noise and signal, unlike the proposed MSTC-PANet. These models necessitate remapping through the linear layer to differentiate between the two, and the overlapping features can adversely influence one another, thereby diminishing classification accuracy. Additionally, for the CLDNN, the features are entirely overlapping, rendering them indistinguishable.

### 4.4. Complexity Analysis

Table 4 shows the impact of different numbers of TCPA modules and the convolution kernel size of the time-scale convolution layer corresponding to each TCPA module on the performance of our proposed MSTC-PANet. In addition, the performance comparison of our model with other models is shown, including sensing error, number of parameters, and Floating Point Operations (FLOPs).

The table indicates that the sensing error in the test dataset decreases as the number of TCPA modules increases. Furthermore, for a fixed number of TCPA modules, an increase in the convolution kernel size employed by the TCPA module is associated with a reduction in sensing error. Our analysis reveals that the computational complexity of the model exhibits a linear relationship with the number of TCPA modules, with the majority of this complexity arising from the parallel structure component. Additionally, while the computational complexity of our model is not superior to that of the DSS model, it demonstrates advantages over the DSS model in terms of parameter count and sensing error, and it significantly outperforms both CLDNN and STFT-CNN. Consequently, to achieve a balance among computational complexity, sensing error, and parameter count, we have opted to implement three TCPA modules, with convolution kernel sizes of 3, 5, and 7 for the convolution layers responsible for scale convolution in each module. Ultimately, our model exhibits a distinct advantage over traditional deep learning approaches and provides a comprehensive investigation into the internal relationships across time scales, as well as the interactions among multiple distinct time scales. This research underscores the intrinsic connections between different time scales in time series data and highlights the complementary nature of these varying time scales.

In conclusion, our proposed MSTC-PANet demonstrates superior performance compared to other models. Furthermore, the complexity of the model can be adjusted by customizing the sampling length and adjusting the quantity of parallel TCPA modules to achieve a desired balance between classification speed and precision.

## 5. Conclusions

This paper investigates the relationship between various time scales in time series data and extends the method used for time prediction to the domain of spectrum sensing, where it has yielded impressive results. The proposed parallel structure, MSTC-PANet, effectively extracts correlations between different time scales in the spectrum sequence and is applicable to both digital and analog modulation. The plug-and-play TCPA module is specifically designed to address the characteristics of time series data in spectrum communication. It integrates residual connections and a self-attention mechanism, enabling it to extract features within a single sequence as well as the correlations between them. Furthermore, acknowledging the interdependence of performance across different time scales, we have developed a parallel structure comprising multiple TCPA modules that operate concurrently. This structure aggregates the final outputs of each TCPA module, allowing MSTC-PANet to capture correlations across multiple time scales simultaneously. Numerical results indicate that the sensing error of MSTC-PANet is superior to that of existing representative models. In future work, we plan to explore the integration of MSTC-PANet into multi-band sensing scenarios and optimize the FLOPs to enhance the effectiveness of spectrum situational awareness.

## Figures and Tables

**Table 1:** Parameters of MSTC-PANet.

Input: IQ Matrix (Dimension: bs × 32 × 128)	
**Layers**	**Kernel Size and Output**
Cnn1d-init	(1 × 3)@32 and (bs, 32, 128)
TCPA-01	Output1 (bs, 64, 128)
TCPA-02	Output2 (bs, 64, 128)
TCPA-03	Output3 (bs, 64, 128)
Feature Aggregation	Output1 + Output2 + Output3
Classifier	2

**Table 2:** Parameters of TCPA Module.

Input: X (Dimension: bs × 32 × 128)	
**Layers**	**Kernel Size and Output**
TS-Conv1d	(1 × 3)@32 and D (bs, 32, 128)
Conv1d-1	(1 × 3)@64 and (bs, 64, 128)
Conv1d-2	(1 × 3)@64 and (bs, 64, 128)
Multi-head Attention	$\hat{D}$ (bs, 64, 128)
Concatenate	D + $\hat{D}$
Output	(bs, 64, 128)

**Table 3:** Parameters of Data Generation.

Parameters	Values
Sampling rate	200 kHz
Samples per symbol	8
Sample length	64, 128
SNR range	[−25, 5] dB
Maximum CFO and SD	500 Hz and 0.01
Maximum SRO and SD	50 Hz and 0.01
Channel environment	Rayleigh fading and Gaussian noise

**Table 4:** Complexity Comparison.

Model	Number of TCPA	Respective Kernel Size	Parameters	Sensing Error (−15 dB)	Flops
MSTC-PANet	1	3	77,378	0.092	7,790,597.0
	1	7	81,474	0.0639	8,314,885.0
	2	5, 7	144,226	0.0365	16,310,277.0
	3	3, 5, 7	204,930	0.0285	24,043,525.0
	4	3, 5, 7, 9	271,778	0.0162	32,563,205.0
DSS	/	/	550,722	0.089	1,745,157.0
CLDNN	/	/	1,073,434	0.312	11,620,101.0
STFT-CNN	/	/	16,868,610	0.214	656,441,866.0

## Data Availability

The original contributions presented in this study are included in the article; further inquiries can be directed to the corresponding author.

## Abbreviations

The following abbreviations are used in this manuscript:

CR	Cognitive Radio
PU	Primary User
IQ	In-phase and Quadrature Component Signals
t-SNE	t-Distributed Stochastic Neighbor Embedding
ROC	Receiver Operating Characteristic Curve
TCPA	Temporal Correlation Perceptual Attention
MATC-PANet	Multi-Scale Time-Correlated Perceptual Attention Model
